# The effects of trans fat diet intake on metabolic parameters and pancreatic tissue in offspring of prenatal bisphenol A exposed rats

**DOI:** 10.1038/s41598-023-36043-1

**Published:** 2023-06-08

**Authors:** Hala Abulehia, Noor Shafina Mohd Nor, Siti Hamimah Sheikh Abdul Kadir, Mardiana Abdul Aziz, Sarah Zulkifli

**Affiliations:** 1 Institute of Medical Molecular Biotechnology (IMMB), Faculty of Medicine, Universiti Teknologi MARA (UiTM), Cawangan Selangor, Kampus Sungai Buloh, Jalan Hospital, 47000 Sungai Buloh, Malaysia; 2 Department of Paediatrics, Faculty of Medicine, Universiti Teknologi MARA (UiTM), Cawangan Selangor, Kampus Sungai Buloh, Jalan Hospital, 47000 Sungai Buloh, Malaysia; 3 Institute for Pathology, Laboratory and Forensic Medicine (I-PPerForM), Universiti Teknologi MARA (UiTM), Cawangan Selangor, Kampus Sungai Buloh, Jalan Hospital, 47000 Sungai Buloh, Malaysia; 4 Department of Biochemistry and Molecular Medicine, Faculty of Medicine, Universiti Teknologi MARA (UiTM), Cawangan Selangor, Kampus Sungai Buloh, Jalan Hospital, 47000 Sungai Buloh, Malaysia; 5 Department of Pathology, Faculty of Medicine, Universiti Teknologi MARA (UiTM), Cawangan Selangor, Kampus Sungai Buloh, Jalan Hospital, Sungai Buloh, 47000 Selangor, Malaysia

**Keywords:** Biochemistry, Endocrinology

## Abstract

Bisphenol A (BPA) is a plasticiser used in the manufacturing of many products and its effects on human health remain controversial. Up till now, BPA involvement in metabolic syndrome risk and development is still not fully understood. In this study, we aimed to investigate the effect of prenatal BPA exposure with postnatal trans-fat diet intake on metabolic parameters and pancreatic tissue histology. Eighteen pregnant rats were divided into control (CTL), vehicle tween 80 (VHC), and BPA (5 mg/kg/day) from gestational day (GD) 2 until GD 21, then their weaning rat’s offspring were fed with normal diet (ND) or trans-fat diet (TFD) from postnatal week (PNW) 3 until PNW 14. The rats were then sacrificed and the blood (biochemical analysis) and pancreatic tissues (histological analysis) were collected. Glucose, insulin, and lipid profile were measured. The study has shown that there was no significant difference between groups with regard to glucose, insulin, and lipid profiles (p > 0.05). All pancreatic tissues showed normal architecture with irregular islets of Langerhans in TFD intake groups compared to offspring that consumed ND. Furthermore, the pancreatic histomorphometry was also affected whereby the study findings revealed that there was a significant increase in the mean number of pancreatic islets in rats from BPA-TFD group (5.987 ± 0.3159 islets/field, p = 0.0022) compared to those fed with ND and BPA non-exposed. In addition, the results have found that prenatal BPA exposure resulted in a significant decrease in the pancreatic islets diameter of the BPA-ND group (183.3 ± 23.28 µm, p = 0.0022) compared to all other groups. In conclusion, prenatal BPA exposure with postnatal TFD in the offspring may affect glucose homeostasis and pancreatic islets in adulthood, and the effect may be more aggravated in late adulthood.

## Introduction

Bisphenol A (BPA) is one of the prominently reported endocrine disruptor chemicals (EDCs). BPA is an organic substance that is widely used in polycarbonate plastic manufacture and epoxy resins including the packaging of food such as canned foods, baby feeding bottles, and frozen food^1^. Numerous studies have measured BPA in different concentrations in blood, urine, placental tissue, follicular fluid, amniotic fluid, breast milk as well as cord serum blood for pregnant women^1,2^. Several studies have shown that BPA can be transmitted directly via the placenta into embryos^2,3^. Since the foetuses possess immature livers and poor drug-metabolizing processes, these could give rise to deleterious impacts of BPA on the foetuses^4^. According to epidemiological and animal findings, prenatal BPA exposure affects metabolic health and increases the risk of obesity and diabetes features in adulthood^5–7^.

An animal experiment was performed to investigate in-utero BPA treatment (0.5 or 50 µg/BPA/kg); in Fischer344 rat offspring, which interestingly exhibited that very low BPA dosage exposure (0.5 µg/BPA/kg) led to an increase in insulin secretion, while a dose of 50 µg/BPA/kg reduced insulin secretion in the mothers and offspring^8^. In-utero BPA exposure (1 µg/BPA/kg) from gestation day (GD) 7.5 to GD 16.5 has been associated with increased levels of glycogen and liver triglyceride (TG), with notably increased blood glucose and insulin concentrations as well as an increase in the serum glucose measured by intraperitoneal-glucose-tolerance-test (IPGTT) and intraperitoneal-insulin-tolerance-test (IPITT) in male offspring aged 14 weeks but not female^9^. Ma et al. showed a significant increase in the serum insulin levels, and a decrease in the insulin sensitivity index (ISI) in male Wistar rats offspring treated with BPA (50 μg/kg/day) relative to the control rats at week 21^5^.

Additionally, human research has revealed that exposure to prenatal BPA may be related to impaired glucose and lipid metabolism. According to a Chinese prospective birth cohort research, a medium BPA level in pregnant mothers was correlated with an increase of 0.36 mmol/L in blood glucose levels in 2-year-old boys^10^. Moreover, a birth-cohort pharmacoepidemiologic study performed on 537 Mexican–American (mother–child pairs) exhibited that boys had an increase in the leptin level related to an increase in BPA levels in the urine in late pregnancy, while urinary BPA levels in early pregnancy (13 weeks) were correlated with increased adiponectin concentrations in girls at 9-year-old^11^.

Recently, accumulating evidence suggests that prenatal BPA exposure has a critical reflection in the epidemic of type 2 diabetes mellitus (T2DM) and obesity^12,13^. Furthermore, findings showed exposure to BPA in developmental periods in-utero, prenatal, and postnatal may lead to the adult phenotype of T2DM, obesity, and tumours^14^.

Based on the finding of epidemiological studies, the intake of trans-fatty acids (TFAs) has been linked with an enhanced threat of cardiovascular diseases (CVD) and metabolic disorders including obesity, insulin resistance (IR), and hepatic disorders^15–18^. TFAs are unsaturated fatty acids with a single double bond at least in the trans-geometric isomerism^19^. World Health Organization (WHO), Food and Drug Administration (FDA) of the USA, and the UK Faculty of Public Health recommended the elimination of TFAs from the diet to decline mortality from coronary heart disease^20,21^.

There are studies (Wei et al., Ding et al. and García-Arevalo et al.) which demonstrated that the high-fat diet (HFD) aggravates metabolic disorders caused by BPA exposure^22–24^. Zulkifli et al. reported the effect of in-utero BPA exposure on metabolic parameters and small intestine morphology in normal diet and trans-fat diet (TFD) offspring group^25^. However, the effect of prenatal BPA exposure on biochemical parameters and pancreas histology response to postnatal TFD intake is not well reported. To the best of our knowledge, this is the first study to investigate whether the disruptions programmed by prenatal BPA exposure could aggravate changes on biochemical parameters and pancreases histology response to postnatal trans-fat diet (TFD) intake. Furthermore, this knowledge will create more awareness among researchers to further understand the impact of EDCs such as BPA and the consumption of TFD in the development of non-communicable diseases such as cardiovascular disease, diabetes and obesity.

Therefore, we hypothesized that in-utero exposure to BPA increases the effect of postnatal TFD on metabolic parameters in the offspring. In our study, pregnant Sprague–Dawley (SD) rats were treated with 5 mg/kg/day of BPA, and weaning male offspring were fed either normal diet (ND) or TFD till postnatal day (PND) 100. We then focused on determining the impact of postnatal TFD intake in combination with in-utero BPA-exposed offspring on blood glucose, insulin, and lipid profile levels, and the effect on pancreatic tissue histology in the adult rat offspring.

## Research methodology

### Rats care and treatment

**Figure 1:**
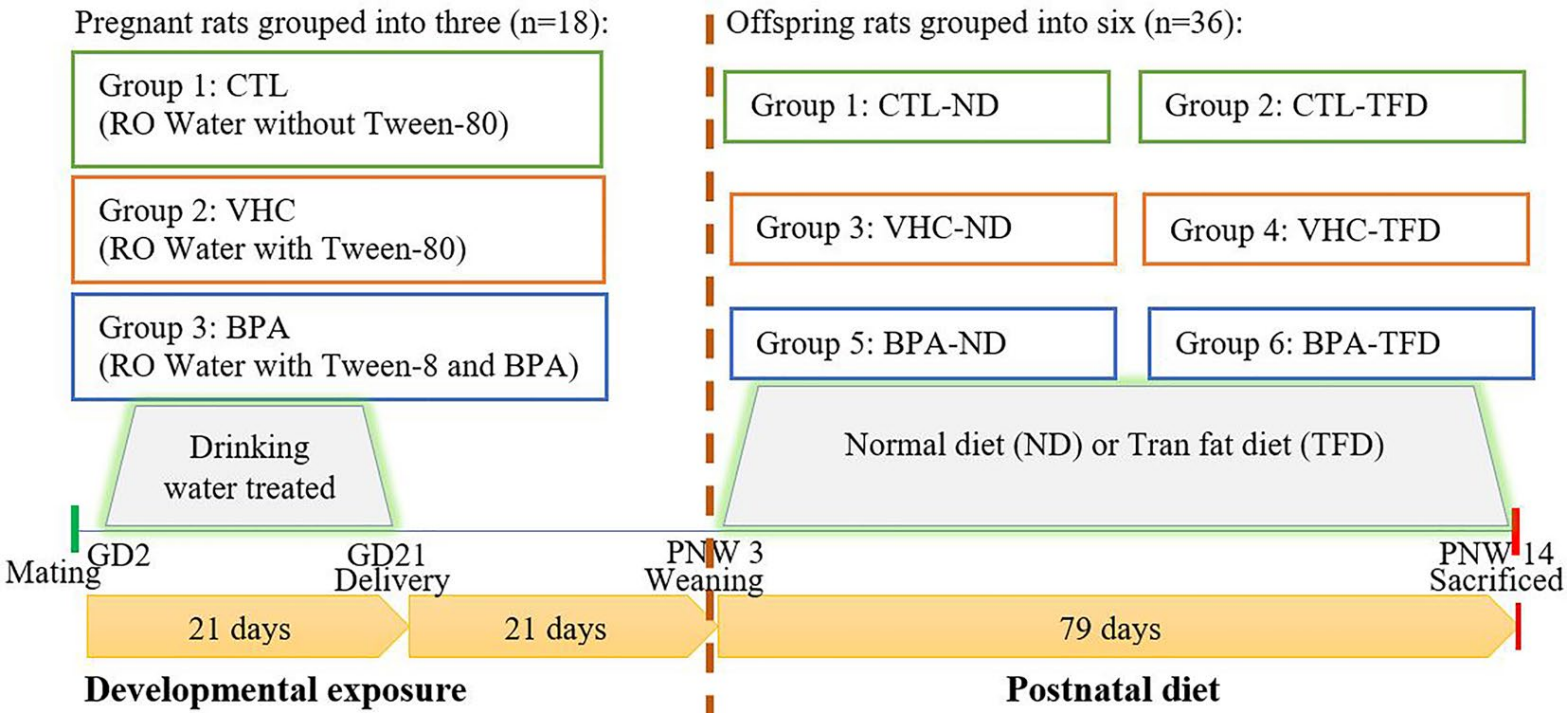
Study design.

All experimental protocols and methods were carried out in accordance with relevant guidelines and regulations and were approved by the Laboratory of Animal Care Unit (LACU) and the ethical rules and standards of the Committee on Animal Research and Ethics, Faculty of Medicine, Universiti Teknologi MARA (UiTM), Sungai Buloh, Selangor where the present research was conducted under protocol approval number UiTM CARE: 294/2020; date of approval: 7 February 2020). Furthermore, all animal experiment protocols and methods were reported in accordance with ARRIVE (Animal Research: Reporting of In Vivo Experiments) guidelines (https://arriveguidelines.org). Female SD (10 weeks) were randomly mated with the male rats (12 weeks) with a 1:1 ratio. The GD 0 was assigned when the white vaginal plug was observed^26^. The gravid mother SD rats were separated randomly into three; control (CTL), vehicle (VHC) (tween 80 drinking water was prepared in 0.25% of final volume), and BPA (5 mg/kg/day) groups (n = 6/groups) which begin on GD 2 till the end of pregnancy. Mothers were treated with BPA or tween 80 through their drinking water to minimise stress in pregnant rats. In all pregnant dams, the body weight (BW), waist circumference (WC), water and food intake were measured on GD 2, 7, and 14. After weaning on the postnatal week (PNW) 3, male pups were randomized and transferred into new cages (6 offspring rats/ group). We selected males rather than females because males were generally the most common sex in rodent research due to cyclical hormonal fluctuations of females and gender differences in metabolic homeostasis, diabetes, and obesity^27^. Meanwhile, many studies have shown that in-utero exposure to BPA has exhibited a sex-specific effect. Besides, animal studies had demonstrated that developmental exposure to BPA led to impaired glucose and lipid homeostasis, induced obesity and increased food intake in adult males, more significantly than in female offspring. This suggests that BPA can lead to more diverse metabolic impairment in males compared to in female offspring^9,24,28,29^. Physiological parameters were estimated for all groups from PNW 3 until 13. Each offspring was supplied with either ND standard rat chow diet (GoldCoin, Port Klang, Malaysia) or TFD (Research Diets Inc, New Brunswick, NJ, USA). The diets were prepared as pellets and stored at 4 °C. The rats had ad libitum access to the food. The TFD constituted 25% kcal fat; the most prominent kinds of trans-fat were elaidic acid as shown in Table 1. At PNW 14, blood samples were taken by cardiac puncture after the adult rats' offspring were deeply anaesthetized by giving sodium pentobarbital (≥ 150 mg/kg) intraperitoneally. Then, the rats were sacrificed by injecting pentobarbital anaesthesia (90 mg/kg) in the peritoneal region, and the pancreatic tissues were harvested and fixed in 10% formalin for histological analysis (Fig. 1).

**Table 1:** The diet composition.

Diet composition	Normal diet (ND)	Trans-fat-diet (TFD)*
Carbohydrate (kcal%)	64%	55%
Protein (kcal%)	27%	20%
Fat (kcal%)	9%	25%
Energy (kcal/gram)	3.9	4.2

*Rodent diet modification with 12% (w/w) hydrogenated vegetable oil.

### Sampling collection

At the end of the animal work, all rats were euthanized after being fasted overnight. The cardiac puncture was used to get the blood samples and then placed in a plain blood collection tube, which were subsequently used to determine the lipid profile (high-density-lipoprotein cholesterol (HDL-C), low-density-lipoprotein cholesterol (LDL-C), total cholesterol (TC), and triglyceride (TG)) as well as insulin levels (IL). The collected blood was centrifuged at 3000 rpm for 10 min at room temperature (20 to 25 °C) and the serum was stored until analysis at − 80 °C. The rats were dissected and the pancreatic tissue was collected in buffered Neutral 10% formalin for histological analysis.

### Measurements of blood glucose, lipid profile, and serum insulin concentrations

The serum levels of lipid profile (HDL-C, LDL-C, TG, and TC) and fasting blood glucose were performed in the Veterinary Laboratory Service Unit (VLSU), Universiti Putra Malaysia (UPM), Selangor using the BA400 analyzer: Sofware version v5.1 (BioSystems, Barcelona, SPAIN). Insulin concentrations were measured by the Rat/Mouse Insulin ELISA kit (cat. # EZRMI-13K) (Merck, Germany), following the manufacturer’s instructions (the intra-variation coefficient (CV) % was 3.30% (triplicate on the same plate) while the inter-CV% was 5.75%).

### Histological analysis

In this study, pancreatic tissue architecture was determined. Pancreatic tissues were rinsed from blood with phosphate-buffered-saline (PBS) and fixed in 10% formalin (pH 7.4). In the paraffin wax, the tissue was embedded after undergoing processing. Afterwards, the pancreas paraffin-wax blocks were put in a − 20 °C freezer for 30 min before being sectioned into 5 µm thickness using a microtome with a sharp blade (Microm HM355 S Microtome, USA). Subsequently, with hematoxylin and eosin (H&E) the pancreatic sections were stained. The sections were qualitatively (morphology) and quantitatively (morphometry) examined using light microscopy (BX53 Olympus, Japan) equipped with a digital camera. All pictures were taken and interpreted by a histopathologist. The following parameters were tested for quantitative pancreatic analysis^30^:

- The average pancreatic islet counts have been estimated using light microscopy in 10 fields selected randomly in each pancreas section at 10 × objective using the light microscope.
- The pancreatic islet mean diameters were measured in six pancreatic islets per section, for an overall of 36 islets in each group, using a measuring tool in cellSenes Dimension software (Olympus, Japan) based on the equation below:
  $${\text{Mean}}\;{\text{diameter}} = \surd {\text{ L}} \times {\text{B}} \times {\text{magnification}}$$
  (L) is the length and (B) the breadth of the islets.

### Statistical analysis

All measurement data were statistically analysed using GraphPad Prism Edition 9. The Kruskal–Wallis test was used to determine whether or not there is a statistically significant difference between the study groups. p-value < 0.05 means that the difference was considered statistically significant. If the Kruskal–Wallis test is significant, the Mann–Whitney U test was performed to examine the unique pairs (to compare each group to another) for multiple comparisons with correction. Measurement data were represented as mean ± Standard Error of the Mean (S.E.M).

### Ethics approval

The animal work protocol was approved by the Animal Research and Ethics Committee of Universiti Teknologi MARA (UiTM) (approval number UiTM CARE: 294/2020; date of approval: 7 February 2020).

## Results

### Effect of BPA exposure during gestation on physiological parameters

#### Effect of BPA exposure on physiological parameters during gestation in pregnant dams

**Figure 2:**
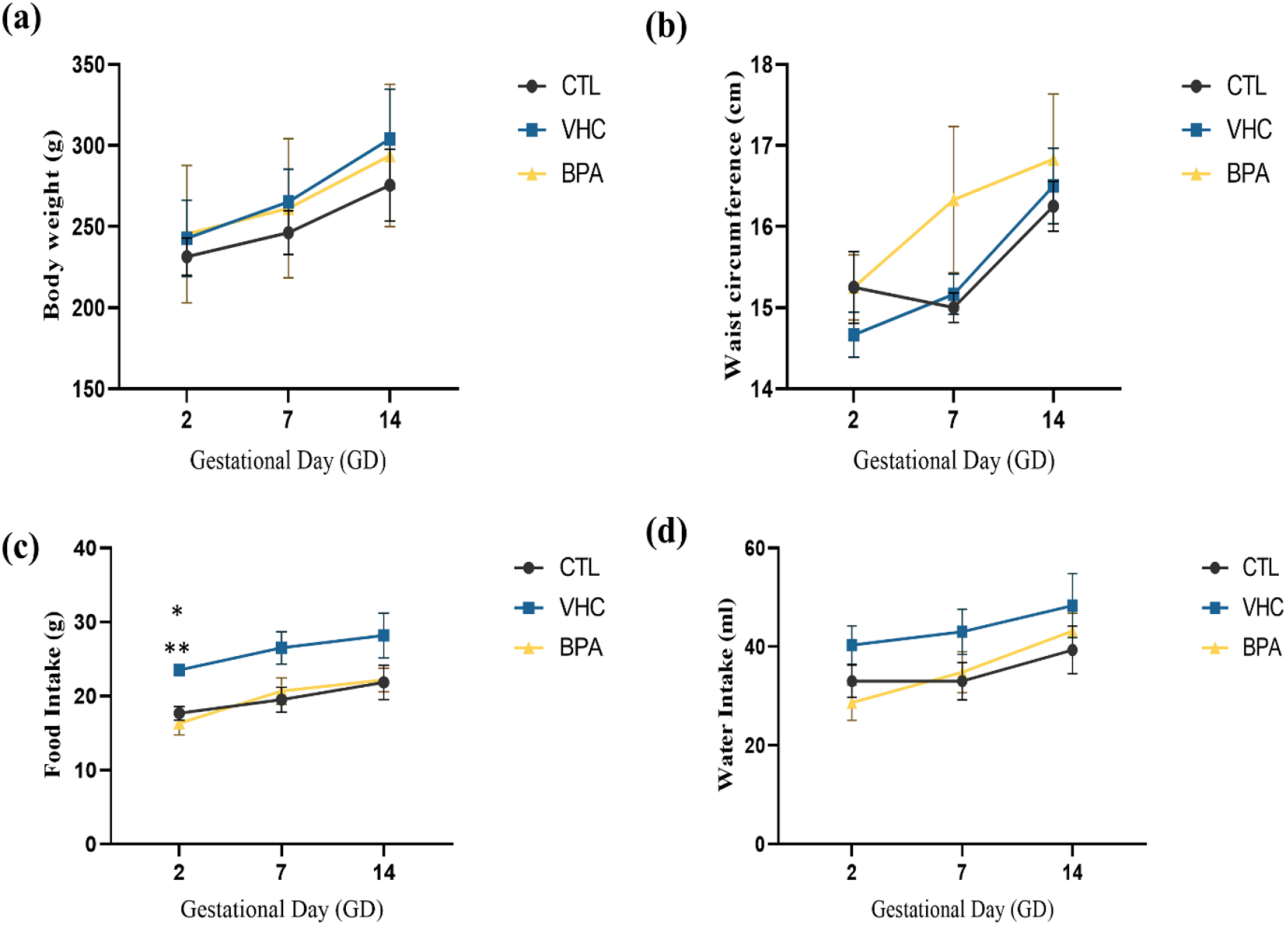
The physiological parameters for pregnant mothers at GD 2,7, and 14. (**a**) Body weight (g), (**b**) waist circumference (cm), (**d**) food consumption (g), and (**c**) water intake (ml). There was no statistically significant difference between all groups of mothers in body weight, waist circumference, and water intake. There was a statistical significance in food intake at GD 2 in the VHC group. The data were represented as mean ± S.E.M for six rats/group (p-value > 0.05).

The study aimed to determine whether exposure to BPA through the gestational period impacted the maternal physiological parameters. The gain in BW and WC as well as the food and water consumption were quantified in the pregnant dams at GD 2, 7, and 14 for all mother groups (Fig. 2). The differences in the BW and WC (p = 0.8286 and P = 0.4179, respectively) were not significant in the BPA-exposed dams during the gestation compared with CTL and VHC controls. Furthermore, BPA exposure did not differentially change dams' water consumption during pregnancy (p = 0.2000) (Fig. 2a–d). In GD 2, the VHC group (23.50 ± 0.764 g) showed significantly higher food consumption than BPA (16.33 ± 1.585 g; p < 0.05) and CTL (17.67 ± 0.919 g; p < 0.01) mother groups (Fig. 2c).

#### Effect of BPA exposure during gestation on newborn’s body weight

**Figure 3:**
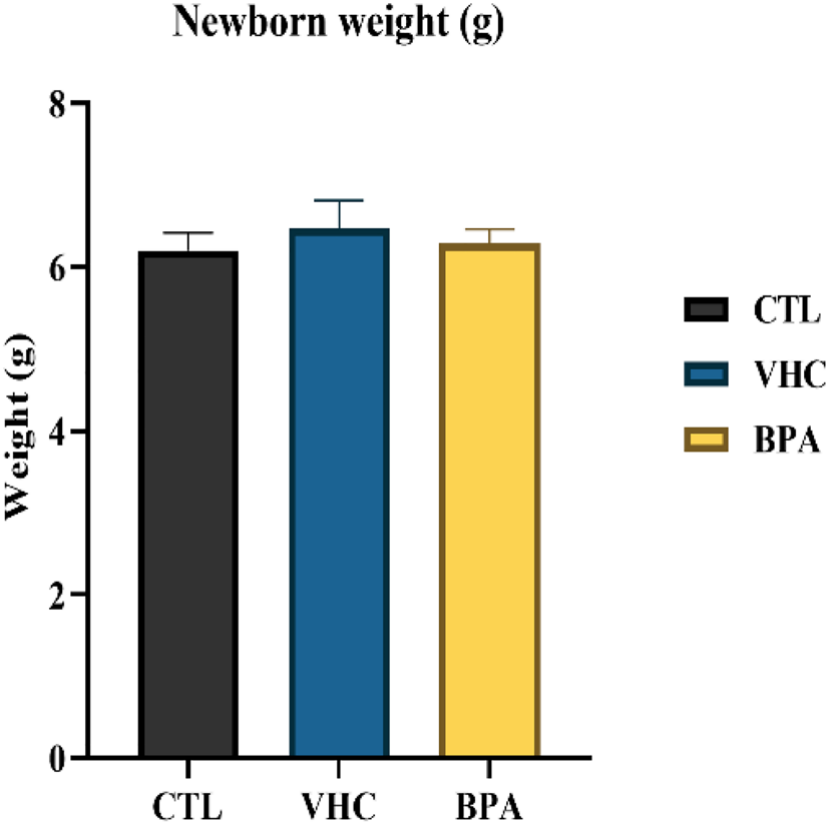
The impact of BPA exposure during pregnancy on neonatal body weight (PND 1). No significant difference is shown between all groups of newborn rat's offspring. The Kruskal–Wallis test was used to determine the differences between BPA and control. The data were represented as mean ± S.E.M for six rats/group. A p-value was considered statistically significant when p < 0.05.

The BW of the newborn was determined to estimate whether prenatal BPA exposure influenced the weight of newborn pups (Fig. 3). The average BW of the pups at PND 1 did not show a significant difference between CTL (6.200 ± 0.2160 g), VHC (6.467 ± 0.3480 g) and BPA-exposed (6.283 ± 0.1759 g) groups (p = 0.5779).

### Effect of prenatal BPA exposure with postnatal trans-fat diet on the anthropometric measurements of 3-and 13-week-old offspring

**Figure 4:**
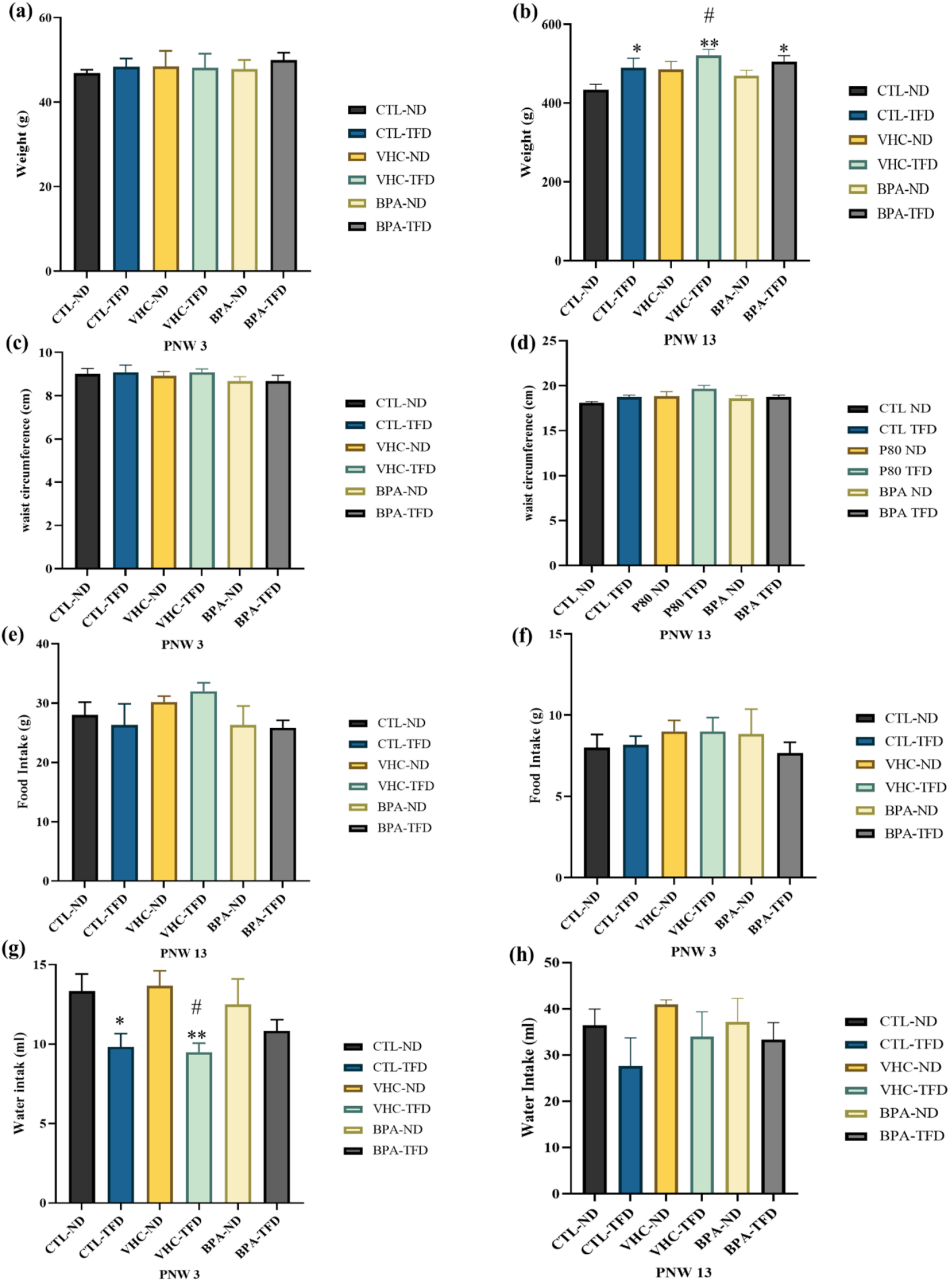
Physiological parameters of 3- and 13-week-old offspring. (**a**) body weight PNW 3, (**b**) body weight PNW 13, (**c**) waist circumference PNW 3, (**d**) waist circumference PNW 13, (**e**) food intake PNW 3, (**f**) food intake PNW 13, (**g**) water intake PNW 3, (**h**) water intake PNW 13. Differences between groups were evaluated by Kruskal–Wallis test. If the Kruskal–Wallis test indicates a statistical significance, subsequently, the Mann–Whitney U test was run to test the unique pairs (p-value < 0.05). Each column is expressed as mean ± S.E.M. for six rats per group. The BW was significantly increased in TFD groups, CTL-TFD vs CTL-ND* (p = 0.0260), VHC-TFD vs CTL-ND** (p = 0.0087), BPA-TFD vs CTL-ND* (p = 0.0152), and VHC-TFD vs BPA-ND# (P = 0.0411) at PNW 13. In contrast, there was a decrease in water intake in the TFD groups at PNW 3. It was significantly decreased in CTL-ND vs VHC-TFD# (p = 0.0108), VHC-ND vs CTL-TFD* (p = 0.0130), and VHC-ND vs VHC-TFD** (p = 0.0065). However, there was a nonsignificant difference in waist circumference and food intake (p > 0.05).

To evaluate whether prenatal BPA exposure and/or postnatal TFD affected the physiological parameters of 3- and 13-week-old offspring, we measured the change in BW and WC as well as the food and water intake at PNW 3 (representative of childhood period), and PNW 13 (representative of adulthood period) in male rat offspring. We determined the mean of the BW of six study groups from delivery until adulthood (n = 6 rat/group). The body weight was significantly increased in TFD groups compared with those treated with a ND at PNW 13 but not PNW 3 (Fig. 4a). There was a significant increase in the BW of CTL-TFD (489.8 ± 24.44 g), VHC-TFD (521.5 ± 15.07 g), and BPA-TFD (505.0 ± 15.62 g) groups compared with CTL-ND (434.2 ± 13.65 g, p < 0.05) group at PNW 13. However, the data showed no significant change in BW in both PNW 3 and 13 in prenatal BPA exposure (Fig. 4a,b). WC and food and water consumption were determined. Briefly, no significant change is shown related to BPA or TFD exposure on the WC as well as food consumption of all study groups in both 3- and 13-week-old male offspring (p > 0.05), (Fig. 4c–f). For water intake, there was a decrease in the amount of water consumed in the TFD group compared with those fed with ND at PNW 3 (Fig. 4g) but not at PNW 13 (Fig. 4h). However, the amount of water was statistically significantly decreased in both CTL-TFD (9.833 ± 0.8333 ml, p = 0.0130) and VHC-TFD (9.500 ± 0.5627 ml, p = 0.0065) but not BPA-TFD (10.83 ± 0.7032 ml, p = 0.0563).

### Effect of prenatal BPA exposure with postnatal trans-fat diet on the metabolic parameters in offspring

We examined the effect of postnatal trans-fat intake combined with prenatal exposure to BPA on metabolic parameters including blood glucose, serum insulin level, and lipid profile in rat offspring at PNW 14.

#### Effect of prenatal BPA exposure with postnatal trans-fat diet on glucose and insulin levels

**Figure 5:**
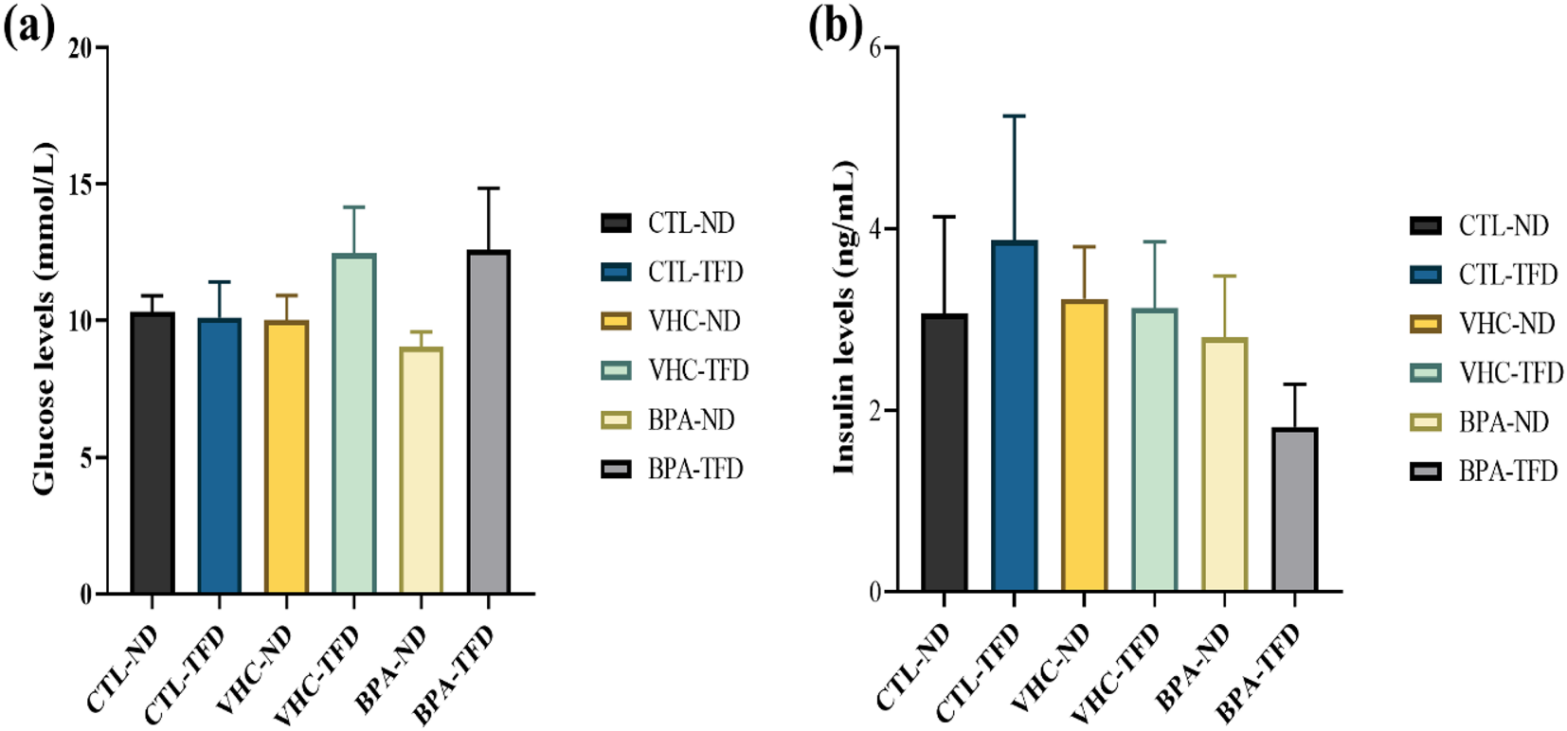
Effect of prenatal BPA and postnatal TFD on (**a**) serum glucose (mmol/L) and (**b**) insulin (ng/mL) concentrations on SD rats at PNW 14. Each column represents mean ± S.E.M. (six rats/group). The result revealed no significant relationship between the study of adult male rats’ offspring.

TFD induced an increase in glucose levels shown in both prenatal vehicle and BPA groups. However, it was not statistically significant between all offspring groups (Fig. 5a). Although serum insulin was increased in CTL-TFD rat's offspring, and a decrease in the levels of insulin in the blood was shown in the BPA-TFD group, however, they were also not significant between all study groups (Fig. 5b).

#### Effect of prenatal BPA exposure with postnatal trans-fat diet on lipid profile levels

**Figure 6:**
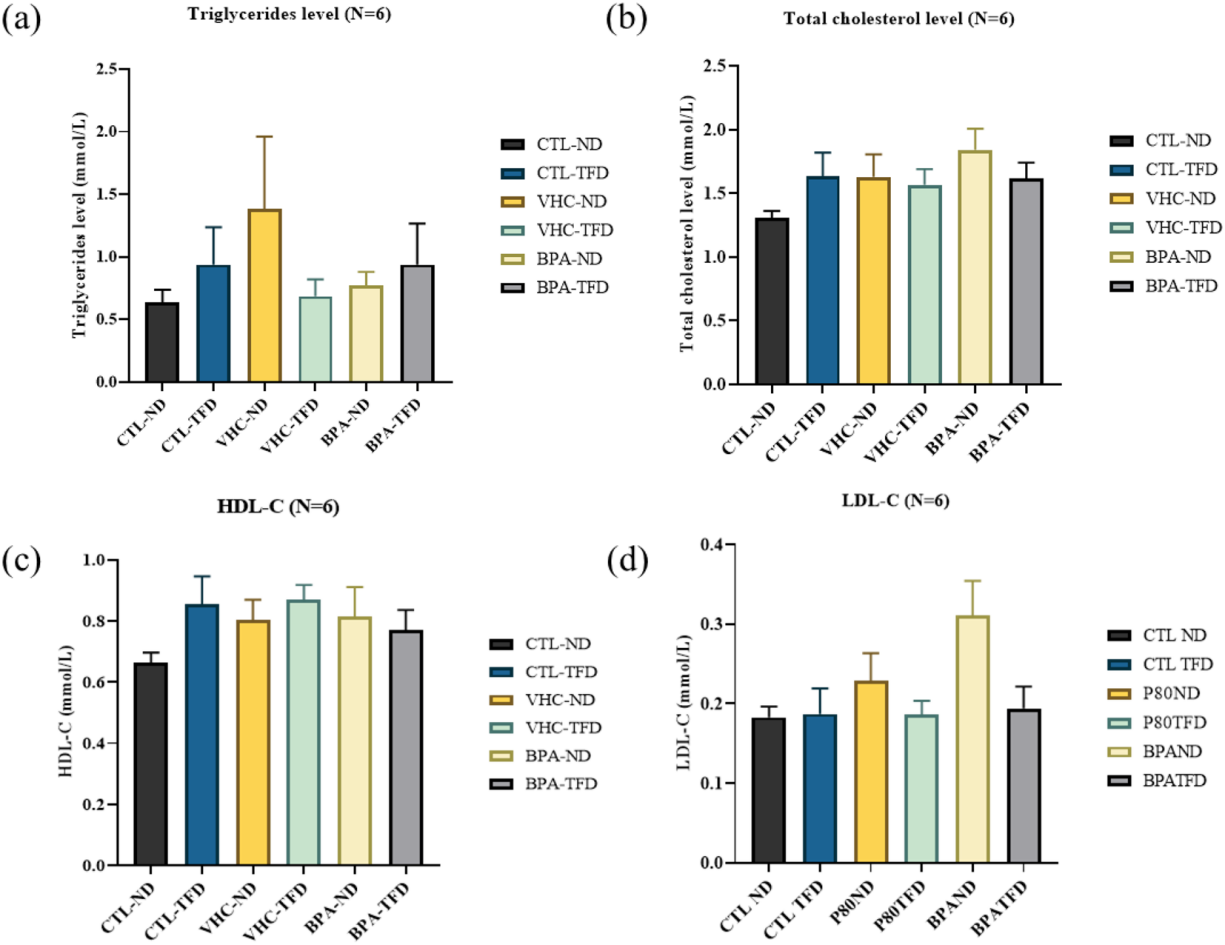
Effect of prenatal BPA and postnatal TFD on TG, TC, HDL-C, and LDL-C serum levels. The column expresses as mean ± S.E.M. (six rats/ group). No statistically significant difference between groups.

As shown in Fig. 6a, postnatal TFD intake led to a slightly increased serum TG level in both prenatal CTL and BPA-treated male rat offspring. However, it was not statistically significant. Interestingly, LDL-C levels were shown to increase only in the BPA-ND group (0.3110 ± 0.04343 mmol/L, p = 0.0823) (Fig. 6b), however, it was not statistically significantly different between groups. BPA prenatal exposure and postnatal TFD revealed no significant impact on TG and HDL-C levels (Fig. 6c,d).

### Effect of prenatal BPA exposure with postnatal trans fat diet on the pancreatic tissue weights in SD rats offspring

**Figure 7:**
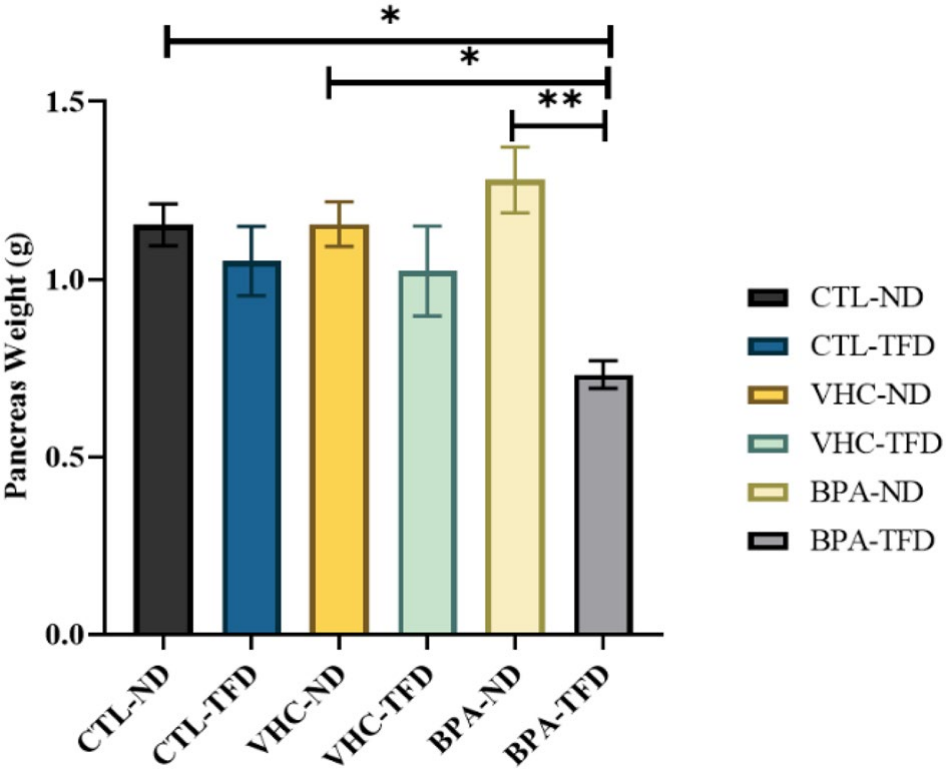
Reduced weight of pancreas tissues in BPA-TFD offspring. *Statistically significant difference exists when compared with BPA-TFD (p < 0.05). The column expressed as mean ± S.E.M.; (n = 6 rat/group).

To study the impact of BPA prenatal exposed rats with postnatal TFD on pancreatic weight, we dissected the rats and harvested the pancreatic tissue. Then, the pancreas of male rat offspring were weighed at PNW 14. There was a higher pancreas weight in BPA-ND, whereas, the lowest pancreas weight was noted in BPA-TFD (Fig. 7).

### Effect of prenatal BPA exposure with postnatal trans-fat diet on histomorphology of the pancreatic tissue in SD rats offspring

**Figure 8:**
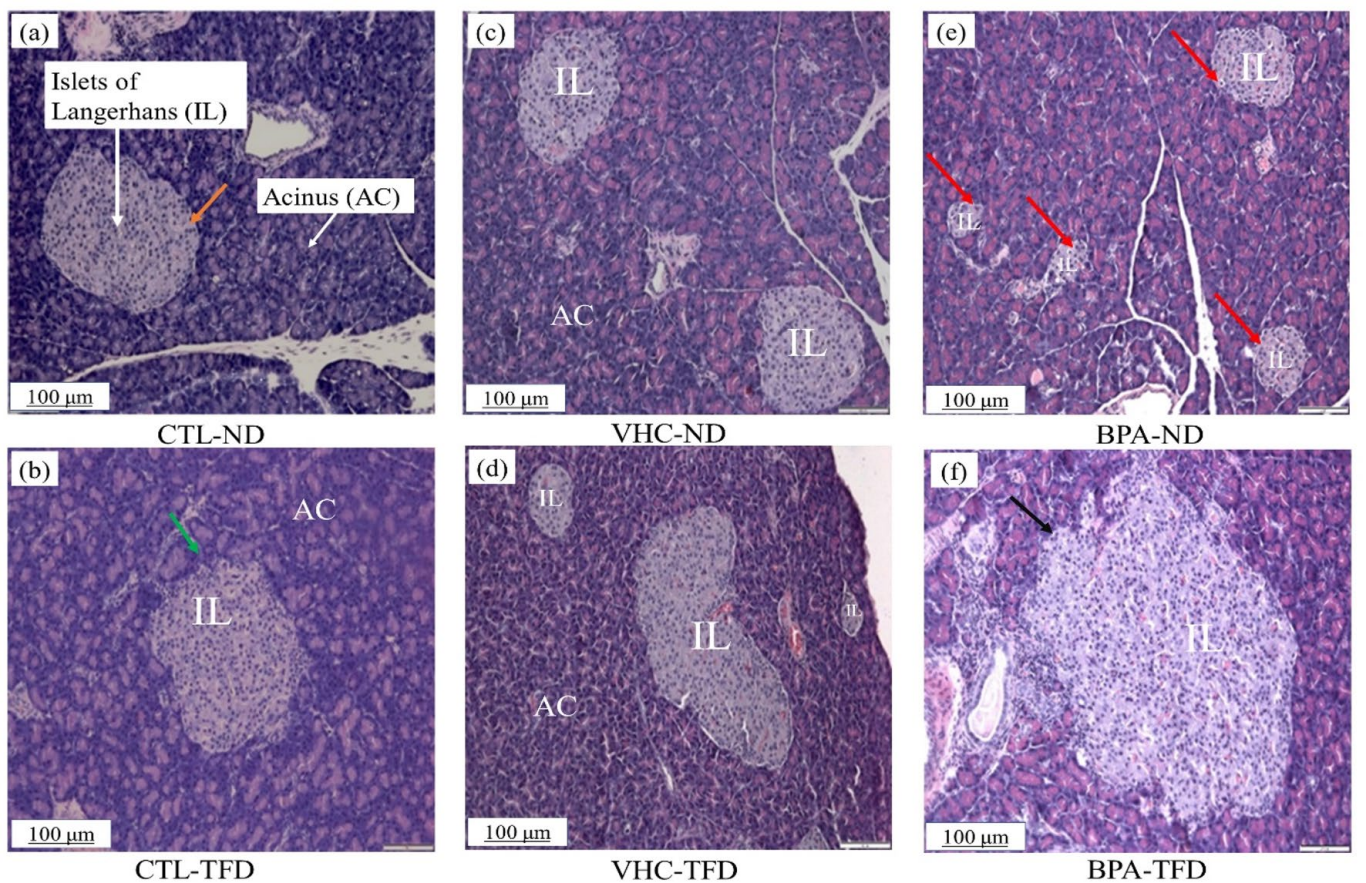
Representative light photomicrographs of the impacts of prenatal exposure to BPA and postnatal TFD on the overall morphology of adult offspring (PNW 14) pancreatic sections from different experimental groups; pancreatic sections were stained with H&E (5 μm). (**a)** Control normal diet group, the pancreatic architecture was normal (CTL-ND). The pancreatic exocrine tissue is composed of packed secretory serous acini arrayed in tiny lobules, which are separated by thin connective tissue septa. The islets of Langerhans are separated from the surrounding acini tissue by a thin fine connective tissue capsule (orange arrow), and it appeared lightly stained than the surrounding acini. (**b**) Control trans-fat diet showing normal architecture of the pancreatic tissue with an irregular border of the islets (green arrow). (**c**) Vehicle (Tween 80) normal diet group showing normal architecture of exocrine and endocrine of the pancreatic tissue. (**d**) Vehicle (Tween 80) trans-fat diet showing normal architecture of the pancreas structure with the irregular border of islets. (**e**) BPA normal diet showing normal pancreas architecture with smaller size islets of Langerhans and abundant number relative to other groups (red arrow). (**f**) BPA-TFD group showing normal pancreas architecture with large size of the islets of Langerhans with the irregular border of islets (black arrow); (n = 6/group). Magnification × 10; scale bar, 100 μm, *IL* islets of Langerhans, *AC* acinus.

To determine the effect of prenatal exposure to BPA with postnatal TFD on the pancreas of adult offspring rats, we analysed the pancreatic tissue sections from each group, and H&E staining was performed. In general, this staining revealed no observable differences in pancreatic tissues' morphology characters in CTL-ND (Fig. 8a) and all other groups (Fig. 8b–f). Nevertheless, the size of Langerhans islets was smaller in BPA-ND (Fig. 8e), whereas, it was larger with an irregular border with TFD treated groups (Fig. 8b,d,f) relative to the control group (Fig. 8a).

### Islets of Langerhans histomorphometry

As we mentioned in histomorphological analysis (3.5) the islets of Langerhans size was affected by BPA and/ or TFD.

**Figure 9:**
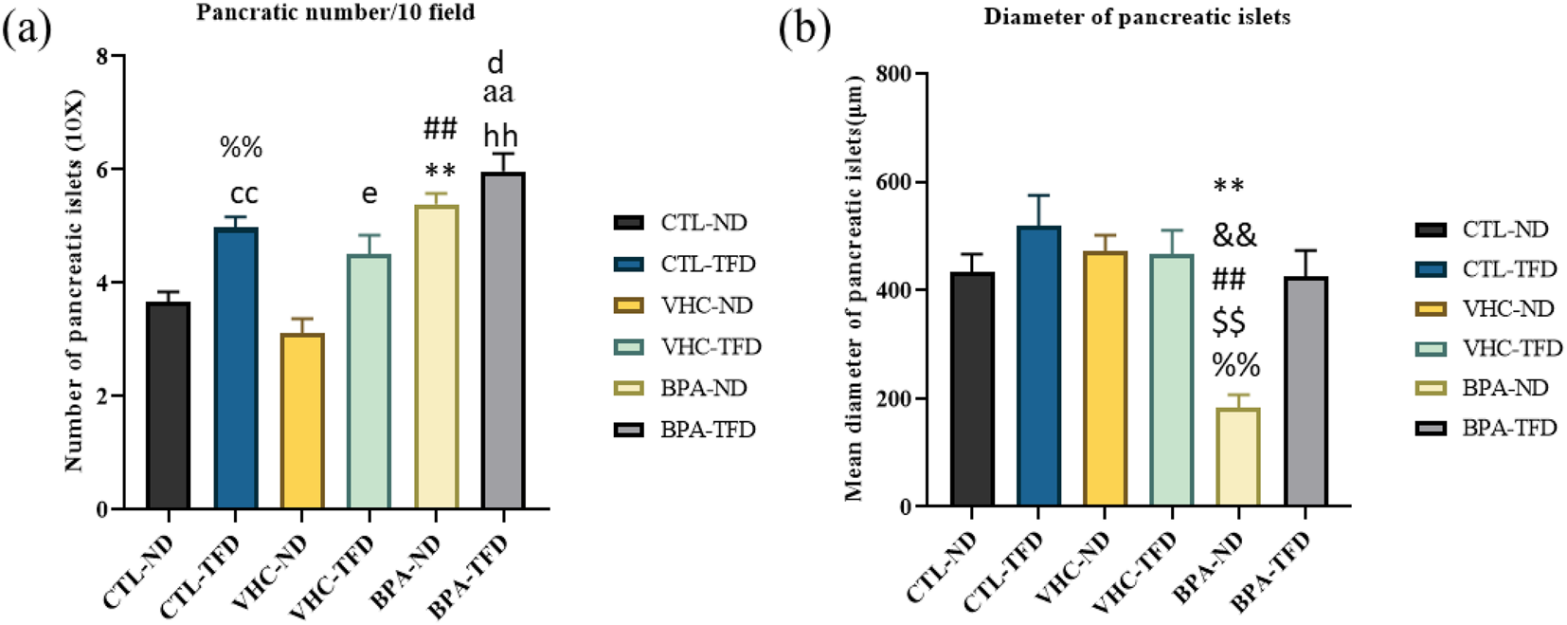
Islets histomorphometry: (**a**) Number of islets in the pancreas for each group expressed as islets number/10 filled for each section 10X. (**b**) The mean of pancreas islets diameter (µm)). Data expressed as mean ± S.E.M.; n = 6/group. A significant decrease was in BPA-ND relative to all other groups. cc CTL-ND versus CTL-TFD, e CTL-ND versus VHC-TFD, **CTL-ND versus BPA-ND, hh CTL-ND versus BPA-TFD, %%VHC-ND versus CTL-TFD, g VHC-ND versus VHC-TFD, ## VHC-ND versus BPA-ND, aa VHC-ND versus BPA-TFD, and d VHC-TFD versus BPA-TFD; && BPA-ND versus CTL-TFD; ## BPA-ND versus VHC-ND; $$ BPA-ND versus VHC-TFD; %% BPA-TFD versus BPA-ND; 1 symbol of significance p < 0.05; 2 symbols of significance p < 0.01.

To define potential changes in pancreatic morphometry following prenatal BPA exposure and postnatal TFD intake, the average pancreatic islet counts was estimated in 10 microscopic fields of 10 × objective lens in each pancreas section. In general, the results have shown a significant increase in the mean of the pancreatic islets number. As compared to rats fed with the normal diet and not exposed to BPA, those treated with prenatal BPA and postnatal TFD consumption showed an increase in the pancreatic islets number. Adult male rat offspring showed an increase in the pancreatic islets number in CTL-TFD (4.967 ± 0.1961 islets/field, p = 0.0022), BPA-ND (5.383 ± 0.1939 islets/field, p = 0.0022) and BPA-TFD (5.987 ± 0.3159 islets/field, p = 0.0022) relative to both controls CTL-ND and VHC-ND (3.667 ± 0.1706 and 3.117 ± 0.2442 islets/field, respectively) (Fig. 9a).

In addition, the mean diameter of pancreatic islets was measured for all groups. Our result showed that prenatal BPA exposure caused a significant decrease in the diameter of pancreatic islets of the BPA-ND group (183.3 ± 23.28 µm, p = 0.0022) compared to all other groups (> 420 µm) (Fig. 9b).

## Discussion

This present study has shown that BPA exposure during pregnancy combined with postnatal TFD (as a metabolic stressor) affected the metabolically active pancreatic tissue, which has a crucial role in glucose homeostasis. In the current experiment, we used the treatment dose of 5 mg/kg/day which is the no-observed-adverse-effect-level (NOAEL) and one-tenth of the lowest-observed-adverse-effect level (LOAEL) of BPA according to the US Environmental Protection Agency (EPA) (50 mg/kg/day) for rodent experiments^31^. Moreover, studies on exposure to repeated-dose of 5 mg/kg/day in rodent models have exhibited impacts on the BW, liver, and kidney^32^. Furthermore, this dose has been shown to affect metabolism including increased BW and altered glucose homeostasis (glucose intolerance, decrease insulin sensitivity and lipid haemostasis) in previous rodent models of gestational exposure^33–35^.

In this current study, we examined the effect of prenatal BPA exposure, and whether this exposure worsens with the intake of a metabolic stressor such as TFD. According to the European Food Safety Authority (EFSA), the amounts of TFA are still high in some foods such as snacks, fast foods, and bakery products in some countries (> 1% of daily energy). In light of the previous studies outcomes such as Wei et al. and García-Arevalo et al., where these studies showed that exposure to BPA affects metabolic processes, and this effect would be accelerated after consuming HFD. Although many studies have provided evidence that BPA is associated with obesity and DM^12,36^, the mechanisms for the occurrence are not yet clear. In addition to changes in the prenatal environment affecting the adult phenotype, there is accumulating evidence that such alteration of development can be exacerbated by the postnatal environment, supporting a “two-hit hypothesis” where a second hit occurs postnatally and can exacerbate a pre-existing condition caused by the first hit earlier in life^37^. Based on what was known about the effects of BPA exposure in combination with a metabolic stressor like a HFD^22^, this study hypothesized that metabolic disorders caused by prenatal BPA (5 mg/kg/day) exposure might cause accelerated metabolic diseases following consumption of postnatal TFD. Recently, a study by Zulkifli et al. investigated the impact of BPA and TFD on obesity and intestinal tissue where they showed that TFD and BPA led to weight gain and changes in intestinal morphology^25^. In the current experiment, we studied the effect of postnatal TFD intake with prenatal BPA mainly on biochemical parameters and on one of the metabolically-active organs, the pancreas, which has a main function in the body's metabolism. Moon et al. showed that long-term exposure to an oral dose of 50 µg/kg/day of BPA stimulated impaired glucose tolerance and reduced insulin sensitivity in mice^38^. In addition, Long et al. indicated that prenatal exposure to BPA significantly increased glucose levels and lipid metabolism alterations detected in 14-week male mice, which were further exacerbated by HFD-induced effects^9^. The current study found that gestational BPA exposure did not affect the physiological parameters of pregnant dams as well as the BW of newborns^39,40^. Similar to the finding by Strakovsky et al. and Xu et al., the results showed that in-utero BPA exposure had no significant change in BW, however, their finding showed that there were impaired lipid or glucose metabolism in some way^41,42^. Here, we demonstrated that prenatal BPA exposure in combination with postnatal TFD (25% kcal fat; with 12% (w/w) hydrogenated vegetable oil) intake from PNW 3 until PNW 14 did not impact the physiological parameters of adult male rat offspring. However, an increase in BW was observed in TFD intake but not BPA exposure, similar to the finding by Zulkifli et al.^25^. These findings are close to a study which showed that maternal BPA exposure did not affect BW at PNW 3 in males and females, however, they observed that there was an increase in the BW when mice consumed HFD. According to another experiment, maternal BPA exposure had no impact on the offspring's BW at PNW 3^39^. However, there was an increase in BW and insulin resistance at PNW 21, which was higher in eight weeks of our adult rats^5^. That may increase the evidence that BPA exposure impact appears in late adulthood. Our findings demonstrated that postnatal TFD intake induced a slight increase in glucose levels in the prenatal vehicle and BPA-exposed rat offspring, although the data showed no significant alteration in the levels between the study groups.

Moreover, in research to examine the impact of TFA, the result has shown no significant change in blood glucose levels of the TFA group compared to the other groups, however, they were higher in insulin levels^43^. Alonso-Magdalena et al. demonstrated that BPA at doses of 10 or 100 μg/kg/day during pregnancy affected glucose tolerance, elevated insulin and leptin levels. This study also reported that the exposure impacted the insulin and glucose tolerance of the male mice offsprings^44^. The conflicting results may be due to several factors, and we suggest that the age of the experimental animals used plays a critical role. We noticed that the glucose tolerance and insulin sensitivity differed according to the age of the animals (17 or 28 week-old)^24^. We expect that the effect of the treatments (BPA and/or TFD) will be more evident in late adulthood, as compared to our study which was done in early adulthood (PNW 14).

During the last decade, studies provided evidence that gestational BPA exposure impaired glucose metabolism in male offspring. In addition, they indicated that there was an alteration in the lipid and glucose haemostasis in the fatty tissue, liver, pancreas, as well as skeletal muscle, and this impairment was similar to the impact in the animals fed with HFD and BPA non-exposed. Furthermore, prenatal BPA exposure increased certain significant HFD effects^24,45,46^. Moreover, experiments indicated that consuming industrial TFA raised the ratio of total serum HDL-C, and increased the cardiovascular disease risk^47^. In a recent review from our group to describe the effect of BPA exposure during pregnancy on biochemical parameters, we noted that there are a few studies which had investigated the effect of the serum lipid profile in the adulthood of first generation offspring^48^. In this study, we measured the serum lipid profile of all rats in six study groups to examine the consequence of in-utero BPA in combination with postnatal TFD intake. Although there was a tendency to increase TG levels in the male rats exposed to BPA and CTL induced with postnatal TFD intake, in addition LDL-C levels were also increased in the BPA-ND group. Our findings showed no statistically significant alteration in serum lipid profile levels between all the study groups at PNW 14. These results are consistent with Diamante et al. who have shown that prenatal BPA exposure in adult male rats significantly increased TG levels. However, there was no statistically significant change in levels of HDL-C, LDL-C, and TC^36^.

In the current experiment, we studied the effect of postnatal TFD intake with prenatal BPA on one of the metabolically-active organs, the pancreas, which has a main function in the metabolism of glucose. Foremost, we measured the pancreas weight of all adult male rat offspring groups. The findings demonstrated a significant decrease in the pancreas weight of the BPA-TFD group compared with CTL-ND, VHC-ND, and BPA-ND. Notably, there was a little decrease in the pancreas weight in the CTL and vehicle groups among the TFD rats. However, there was a significant decrease with a combination of BPA and TFD consumption. One study showed that the mean weights and relative ratios of the pancreas were significantly decreased in diabetic rats than in control rats^49^. This indicates that the BPA-TFD group may be at higher risk of diabetes than the other groups.

We also analysed the pancreatic tissue section stained with H&E and examined the pancreas typical architecture in all study groups. BPA-ND group appeared to have smaller islets of Langerhans size and an abundant number relative to other groups, while BPA-TFD group appeared to have a larger size of the islets of Langerhans with an irregular border of islets. Furthermore, we determined the histomorphometry analysis of the Islets of Langerhans. In both BPA-ND and BPA-TFD, the mean number of pancreatic islets increased significantly relative to CTL-ND and VHC-ND. Another study has investigated the effect of gestational exposure to BPA in the foetus and reported that 5 mg/kg/day of prenatal BPA altered mouse foetal pancreatic development. The study found that prenatal BPA-exposed foetal pancreas had more islet-cell clusters than the control^50^.

These indicated that prenatal exposure to 5 mg/kg/day may affect pancreatic tissue from the foetus until adulthood and may induce alteration in glucose and insulin metabolism in late adulthood. Recent findings reported that BPA had no detrimental alterations in the morphology, pancreatic islet size or the content of insulin in β cells^38^. Although this BPA dose was ten times higher than our study exposure, there was no effect on the pancreas islets. These suggest that BPA exposure during a critical period such the pregnancy increases the risk factor for metabolic disease according to “foetal origins of adult disease” theory. This highlights how detrimental effects throughout the early phases of development, especially during intrauterine life are associated with future physiological disorders in adulthood, which raises the probability of chronic disease development in adulthood^51^.

In this study, the impact of treatments on metabolic parameters and pancreas tissue was investigated. Many studies examined the effect on the organs such as the liver, muscles, fatty tissues and the pancreas^28,52^. Here, we have focused on the pancreas because of its impact on the pathogenesis of metabolic disorders, especially T2DM. The perceived limitation was that this work studied the impact of biochemical parameters and the pancreas tissue on a single timepoint at PNW 14. The insulin content had not been measured in the pancreatic tissue; therefore, we recommend further research to measure insulin in the pancreatic tissue since prenatal BPA exposure and postnatal TFD affected pancreas morphology in this study. The findings of this study can contribute to the literature on the effects of TFD intake on in-utero BPA exposure. The outcome of the study is also important to assist the government to put in place mandatory legislation on TFA levels in the production of food. This can help to reduce non-communicable disorders such as coronary heart disease, T2DM, and obesity. According to EFSA, the amounts of TFA are still high in some foods such as snacks, fast foods, and bakery products in some countries^53^. Besides, the finding of this study is also important to increase the awareness of reducing the usage of BPA products and TFD consumption.

## Conclusion

Many research data have indicated that BPA or TFD can affect insulin and glucose metabolism. However, our study findings showed no statistically significant changes among the study groups related to the serum glucose, insulin, and lipid profile (TG, TC, HDL-C, and LDL-C). Conceivably, the discrepancy in the results was influenced by the difference in the study model, time of treatment exposure, small unpredicted environmental stressors, or the batches of the diet used. However, the study has shown that prenatal BPA with postnatal exposure to TFD is a metabolic stressor affecting the pancreas tissue histomorphometry. This may reflect on the metabolic parameters later in late adulthood. Therefore, we emphasize that there is a need for more future epidemiological and in-vivo research to better understand the effect of both BPA and TFD on different life stages from foetus to late adulthood to better understand the impacts.

## Data Availability

The datasets used and/or analysed during the current study is available from the corresponding author upon reasonable request.

## Abbreviations

ARRIVE: Animal Research: Reporting of In Vivo Experiments
BPA: Bisphenol A
BW: Body weight
CTL: Control
CVD: Cardio-vascular diseases
EDCs: Endocrine disruptor chemicals
EPA: Environmental Protection Agency
FDA: Food and Drug Administration
GD: Gestation Day
H&E: Hematoxylin and eosin
HDL-C: High-density-lipoprotein cholesterol
IL: Insulin levels
IPGTT: Intraperitoneal-glucose-tolerance-test
IPITT: Intraperitoneal-insulin-tolerance-test
ISI: Insulin sensitivity index
LACU: Laboratory of Animal Care Unit
LDL-C: Low-density-lipoprotein cholesterol
ND: Normal diet
NOAEL: No-observed-adverse-effects-level
PBS: Phosphate-buffered-saline
PND: Postnatal day
PNW: Postnatal week
SD: Sprague–Dawley
SEM: Standard error of the mean
T2DM: Type-2 diabetes mellitus
TC: Total-cholesterol
TFAs: Trans-fatty-acids
TFD: Trans-fat-diet
TG: Triglyceride
UiTM: Universiti Teknologi MARA
UPM: Universiti Putra Malaysia
VLSU: Veterinary Laboratory Service Unit
WC: Waist circumference
